# The Potential of Edible Bird’s Nests in Reducing Cardiovascular Disease Risk Factors: A Narrative Review

**DOI:** 10.3390/ijms26104619

**Published:** 2025-05-12

**Authors:** Nina Diyana Rusanuar, Amilia Aminuddin, Adila A. Hamid, Jaya Kumar, Chua Kien Hui, Mohd Kaisan Mahadi, Azizah Ugusman

**Affiliations:** 1 Department of Physiology, Faculty of Medicine, Universiti Kebangsaan Malaysia, Cheras 56000, Malaysia; ninadiyana@ukm.edu.my (N.D.R.); amilia@hctm.ukm.edu.my (A.A.); adilahamid@ppukm.ukm.edu.my (A.A.H.); jayakumar@ukm.edu.my (J.K.); 2 Cardiovascular and Pulmonary (CardioResp) Research Group, Universiti Kebangsaan Malaysia, Bangi 43600, Malaysia; 3 Glyness Industries Sdn Bhd, Cheras 43200, Malaysia; ckienhui@gmail.com; 4 Centre for Drug and Herbal Development, Faculty of Pharmacy, Universiti Kebangsaan Malaysia, Jalan Raja Muda Abdul Aziz, Kuala Lumpur 50300, Malaysia; kaisanmahadi@ukm.edu.my

**Keywords:** antioxidant, cardiovascular disease, diabetes mellitus, dyslipidemia, edible bird’s nest, inflammation, obesity

## Abstract

Cardiovascular disease (CVD) remains a leading cause of mortality worldwide, with dyslipidemia, obesity, diabetes mellitus, and hypertension being major modifiable risk factors. Functional foods with antioxidant and anti-inflammatory properties have gained attention for their potential for reducing CVD risk. Edible bird’s nest (EBN), a functional food rich in bioactive compounds such as sialic acid, lactoferrin, and glycoproteins, has been shown to exhibit antioxidant and anti-inflammatory effects. This review explores the potential of EBN in mitigating CVD risk factors, focusing on its role in improving lipid profiles, managing obesity, and enhancing glucose metabolism. EBN has been shown to improve the lipid profile by regulating the hepatic cholesterol metabolism and gut–liver axis interactions. Additionally, EBN reduces body weight gain and visceral fat accumulation, improves adipokine regulation, and enhances insulin sensitivity, which may collectively support cardiovascular health. Despite promising findings, clinical evidence remains limited. Future research should focus on clinical trials to validate its efficacy, determine optimal dosages, and assess its long-term safety. Additionally, further studies on EBN’s effects on hypertension and its interaction with conventional therapies could enhance its potential role in CVD prevention and management.

## 1. Introduction

Cardiovascular disease (CVD) is a broad term encompassing disorders that affect the heart and blood vessels, including coronary artery disease, stroke, heart failure, and hypertension. CVD remains a significant global health burden as the leading cause of death, accounting for 20.5 million deaths in 2021 [1]. Several modifiable and non-modifiable risk factors contribute to the development of CVD. Genetic predisposition, aging, and male gender are among the non-modifiable risk factors that increase susceptibility to CVD [2]. Major modifiable risk factors include hypertension, diabetes mellitus (DM), obesity, dyslipidemia, and smoking, while sedentary lifestyles, chronic stress, and unhealthy dietary habits further exacerbate the condition [3]. The major modifiable risk factors for CVD, such as hypertension, DM, obesity and dyslipidemia, are associated with increased oxidative stress, atherosclerosis, and chronic low-grade inflammation, all of which contribute to an elevated risk of CVD [4].

The rising number of CVD-related deaths underscores the urgent need for effective management strategies. However, prevention remains the most effective approach for reducing the CVD burden [5]. Emerging research highlights functional foods as a cost-effective and promising alternative for both the prevention and management of CVD, offering cardioprotective benefits through their antioxidant, anti-inflammatory, and lipid-lowering properties [6]. The exploration of functional foods as complementary or alternative approaches to standard therapies is gaining attention, driven by an increasing consumer preference for safer, natural, and cost-effective interventions in cardiovascular health [7]. One of the promising functional foods for cardiovascular health is edible bird’s nest (EBN).

EBN is a renowned traditional Chinese medicine that has been consumed by the Chinese population since the Tang Dynasty [8]. It is widely regarded as a health tonic and has long been used as a nutritional supplement to promote general well-being [9]. Traditionally, EBN has been used to alleviate coughs, improve digestion, enhance the immune system, and provide anti-aging benefits [10,11,12]. China is one of the world’s largest importers of EBN. In 2021, China imported approximately 128.3 tons of EBN from Indonesia, 42.3 tons from Malaysia, and 0.1 tons from Thailand [13].

EBN is produced from the saliva secreted by the insectivorous swiftlets, which are predominantly found in Southeast Asia and the southern regions of China [14]. Two genera of swiftlets known to produce high-quality EBN are *Aerodramus* and *Collocalia* [15]. There are three main types of bird nests: white, black, and red. White nests are composed almost entirely of swiftlet saliva and are typically harvested from swiftlet houses, whereas black nests contain approximately 45–55% feathers and small dried leaves and are collected exclusively from caves. Some nests, which range from a dull orange-red to brownish-red, are occasionally found in caves and swiftlet houses [16,17,18].

The nutrient composition of EBN varies depending on the bird species, geographical location, and harvesting season [19,20]. EBN primarily consists of protein, accounting for 50–55% of its total composition, along with carbohydrates, ash, fats, minerals, and vitamins [21,22]. Notably, EBN contains sialic acid, an important carbohydrate with various biological activities, including anti-inflammatory, antioxidant, anti-viral, and anti-tumor properties [14,23,24]. Numerous preclinical studies have explored the therapeutic benefits of EBN. These studies have demonstrated its antiviral [25], anti-inflammatory [26], antioxidant [9,27,28], and anticancer [14] properties, as well as its neuroprotective effects. Additionally, EBN has been shown to exert cardiovascular protective effects by influencing lipid profiles, coagulation status, cholesterol metabolism, and insulin sensitivity, thereby exhibiting potential antiatherogenic properties [29,30,31].

Diet plays a pivotal role in cardiovascular health by modulating systemic inflammation, lipid metabolism, endothelial function, blood pressure, and body weight [32]. One important dietary-linked metabolic mediator is trimethylamine N-oxide (TMAO), a compound derived from the gut microbial metabolism of choline and carnitine, found in red meat and eggs. Elevated levels of TMAO have been independently associated with an increased risk of atherosclerosis and adverse cardiovascular events [33], supporting the importance of dietary patterns in cardiovascular health. In contrast, plant-based diets and those rich in polyphenols have been shown to lower TMAO levels and reduce cardiovascular risk [34]. Among dietary interventions, nutraceuticals such as grape pomace extract, which contains polyphenols like resveratrol and proanthocyanidins, have demonstrated potent antioxidant and anti-inflammatory properties, contributing to improved vascular function, lipid profiles, and blood pressure regulation [35]. These compounds act through mechanisms such as reducing oxidative stress, enhancing nitric oxide bioavailability, and inhibiting pro-inflammatory pathways [36,37].

Within this context, EBN represents a novel and culturally established nutraceutical with promising cardiovascular benefits. Notably, sialic acid, one of the most studied bioactive compounds in EBN, has shown antioxidant, anti-inflammatory, and antihypertensive effects [9,38,39]. As part of an overall nutraceutical strategy, EBN may serve as a valuable addition to dietary interventions aimed at preventing or managing CVD, particularly in populations seeking natural, food-based alternatives to conventional therapy.

Although the medicinal value of EBN has long been recognized, scientific reviews specifically examining its effects on CVD risk factors remain limited. Existing reviews have primarily focused on the antimicrobial, anti-inflammatory, antioxidant, and cognitive-enhancing properties of EBN, with minimal emphasis on its role in modulating the key modifiable risk factors of CVD. Given that oxidative stress and inflammation are central to the pathophysiology of CVD, the antioxidant and anti-inflammatory properties of EBN may represent a significant potential in mitigating these risks. This review aims to address this gap by evaluating the current evidence on the effects of EBN on major modifiable CVD risk factors, including DM, obesity, hypertension, and dyslipidemia, thereby highlighting its potential as a natural therapeutic strategy for CVD prevention and management.

## 2. Literature Search Strategy for Narrative Review

A structured literature search was conducted as part of this narrative review to identify relevant studies examining the effects of EBN on CVD risk factors. The search was carried out across five databases—PubMed, Scopus, Google Scholar, OVID Medline, and Web of Science—covering publications from 2014 to 2024. The following search terms were used: ‘edible bird’s nest’ AND (cardiovascular OR cardiovascular disease OR hypertension OR dyslipidemia OR diabetes OR obesity OR antioxidant OR inflammation). The aim was to capture original articles that investigated the potential of EBN in modulating risk factors associated with CVD. Both preclinical (in vitro and in vivo) and clinical studies were considered. Review articles, editorials, conference abstracts, non-English language publications, and studies not addressing the effects of EBN on CVD-related outcomes were excluded.

**Figure 1:**
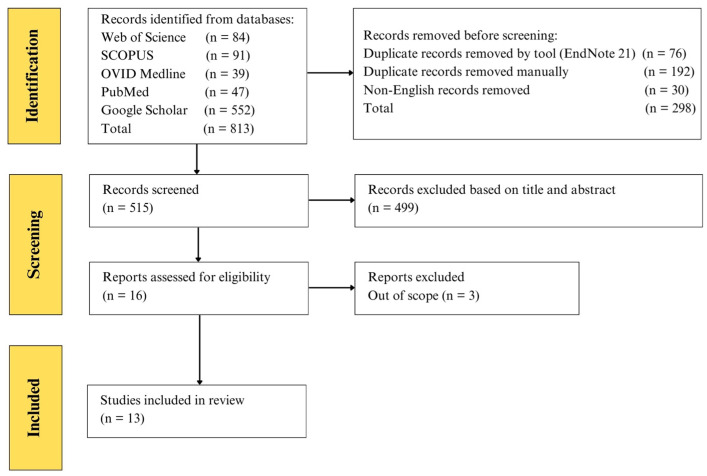
The PRISMA flow chart of the structured literature search conducted as part of the narrative review.

All search results were imported into EndNote version 21 reference management software, which was used to organize citations and automatically identify and remove duplicate records. The remaining articles were then screened by the authors in two stages: (1) title and abstract screening and (2) full-text review to confirm relevance based on the predefined scope of this review. A total of 515 articles were initially retrieved, of which 13 were deemed relevant and included in this narrative review (Figure 1). As this is a narrative review rather than a systematic review, formal quality assessment tools or systematic selection procedures (such as PRISMA guidelines, dual reviewers, or a structured risk of bias assessment) were not applied. The study selection was guided by the relevance to the topic as judged by the authors.

## 3. Effects of EBN on Dyslipidemia and Lipid Metabolism

Dyslipidemia is a key cardiometabolic risk factor that leads to the development of CVD [40]. Clinically, dyslipidemia is characterized by elevated serum levels of triglycerides (TG), total cholesterol (TC), and low-density lipoprotein cholesterol (LDLc), alongside reduced levels of high-density lipoprotein cholesterol (HDLc) [41]. Dyslipidemia increases the CVD risk primarily by promoting atherogenesis, a process exacerbated by chronic inflammation and oxidative stress. The accumulation of LDL and triglyceride-rich lipoproteins in the arterial walls triggers inflammatory responses, leading to the formation of oxidized LDL (OxLDL) [42]. OxLDL is both pro-inflammatory and pro-atherogenic, further increasing reactive oxygen species (ROS) production [29].

EBN supplementation has been shown to improve lipid profiles by reducing serum TC, TG, and LDLc levels while increasing HDLc levels in various diet-induced hypercholesterolemic animal models. This includes rats and mice fed a high-fat diet [30,42,43,44,45], as well as rabbits and rats fed a combined high-fat and high-cholesterol diet [46,47]. Furthermore, these studies reported a decline in oxLDL, indicating EBN’s ability to reduce oxidative stress-related lipid peroxidation [30,44]. Similar findings were observed in atherosclerotic New Zealand white rabbits, where EBN supplementation lowered atherogenic indices, which are markers of cardiovascular risk [46]. These findings highlight EBN’s potential in mitigating hypercholesterolemia and atherosclerosis progression.

The lipid-lowering effects of EBN are mediated through multiple molecular mechanisms. One key mechanism involves the regulation of hepatic cholesterol metabolism. Studies have shown that EBN supplementation increases the expression of the low-density lipoprotein receptor (*LDLR*), lectin-like oxidized LDL receptor-1 (*LOX-1*), and cluster of differentiation 36 (*CD36*), all of which enhance cholesterol uptake and clearance. Additionally, EBN upregulates genes responsible for cholesterol efflux and bile acid metabolism, including ATP-binding cassette transporter A1 (*ABCA1*), lecithin-cholesterol acyltransferase (*LCAT*), and cholesterol 7 alpha-hydroxylase (*CYP7A1*), while downregulating proprotein convertase subtilisin/kexin type 9 (PCSK9), apolipoprotein B (*APOB*), and 3-hydroxy-3-methylglutaryl-CoA reductase (*HMGCR*), thereby reducing hepatic cholesterol biosynthesis [44,46].

Additionally, EBN has been shown to inhibit key enzymes involved in cholesterol synthesis and lipid digestion. In vitro and in vivo studies demonstrated that EBN supplementation reduced HMGCR and lipase activity, which are essential in cholesterol biosynthesis and dietary fat absorption, respectively [47]. These enzyme inhibitory effects further reinforce EBN’s potential as a multi-target intervention for dyslipidemia. Beyond hepatic regulation, EBN also modulates adipokine levels, which are critical in lipid metabolism and energy homeostasis. A study reported that EBN supplementation increases adiponectin levels while reducing leptin levels, leading to an improved insulin sensitivity and lipid profile [30]. Furthermore, EBN appears to exert estrogen-like effects in lipid regulation. In ovariectomy-induced menopausal rats, where estrogen deficiency disrupts cholesterol metabolism, EBN supplementation significantly improved lipid profiles, suggesting its potential role in hormone-regulated lipid metabolism [48].

Recent findings have also highlighted EBN’s role in the gut–liver axis, which plays a crucial role in lipid metabolism. EBN supplementation in high-fat-diet-induced obese mice resulted in modifications of the gut microbiota, increasing the beneficial bacteria associated with bile acid metabolism and lipid absorption. This alteration led to enhanced hepatic bile acid excretion and reduced intestinal bile acid reflux, further contributing to cholesterol clearance and lipid homeostasis [42]. These findings suggest that EBN exerts its lipid-lowering effects not only through hepatic transcriptional regulation but also via gut microbiome-mediated mechanisms.

Several bioactive compounds present in EBN have been suggested to contribute to its lipid-lowering effects, including sialic acid, lactoferrin, ovotransferrin, bakuchiol, and dehydrolindestrenolide [42,43,44,46,47]. These compounds have been previously linked to cholesterol metabolism, lipid regulation, and anti-inflammatory properties, which may explain EBN’s effectiveness in managing dyslipidemia. Sialic acid is one of the most well-documented compounds in EBN, known for its anti-inflammatory, antioxidant, and metabolic-regulating properties [42,49]. Studies have suggested that sialic acid plays a role in modulating lipid metabolism, as an increased sialic acid content in LDLc has been associated with enhanced cholesterol clearance and reduced atherogenesis [42]. In other models, sialic acid has been found to enhance HDLc-mediated cholesterol efflux, contributing to improved lipid profiles [50]. This aligns with findings from EBN studies, where EBN supplementation significantly increased HDLc levels while reducing LDLc and atherogenic indices [46].

Lactoferrin and ovotransferrin, both glycoproteins identified in EBN, have been implicated in lipid homeostasis. Lactoferrin has been shown to reduce serum cholesterol levels by modulating *LDLR* expression and inhibiting HMGCR activity, similar to statins [51]. Studies have also demonstrated lactoferrin’s ability to attenuate hepatic lipid accumulation and oxidative stress, making it a potential therapeutic agent for hyperlipidemia and fatty liver disease [52]. Given that EBN supplementation has been linked to an upregulated *LDLR* expression and reduced HMGCR activity, it is plausible that lactoferrin contributes to these effects [53].

Bakuchiol and dehydrolindestrenolide have shown potential lipase-inhibitory and cholesterol-lowering effects [47,54]. Bakuchiol, a bioactive terpenoid, has been reported to suppress HMGCR activity and promote bile acid excretion, reducing overall serum cholesterol levels [55]. This is consistent with a previous study finding, where EBN supplementation significantly decreased *HMGCR* expression and lipase activity [47], suggesting a similar mechanism. Additionally, dehydrolindestrenolide has demonstrated anti-atherogenic properties by modulating lipid uptake and efflux pathways, reinforcing its role in cholesterol metabolism [47].

**Figure 2:**
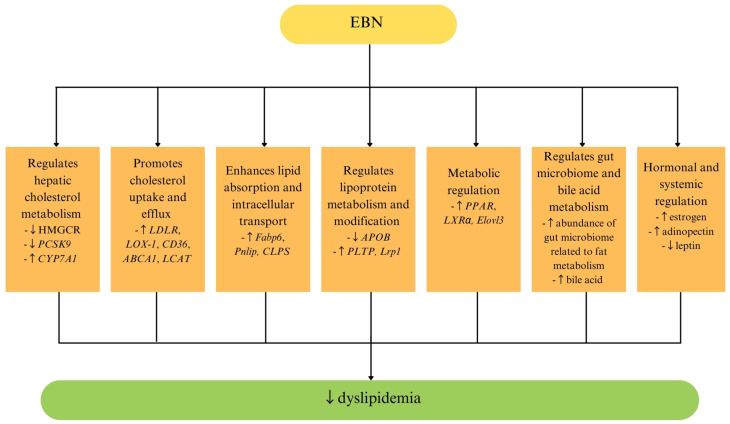
Effects of EBN on dyslipidemia and lipid metabolism. Abbreviations: ↑, increase; ↓, decrease; *ABCA1*, ATP-binding cassette transporter A1; *APOB*, apolipoprotein B; *CD36*, cluster of differentiation 36; *CLPS*, colipase; *CYP7A1*, cholesterol 7 alpha-hydroxylase; EBN, edible bird’s nest; *Elovl3*, elongation of very long chain fatty acids protein 3; *Fabp6*, fatty acid binding protein 6; HMGCR, 3-hydroxy-3-methylglutaryl-CoA reductase; *LCAT*, lecithin-cholesterol acyltransferase; *LDLR*, low-density lipoprotein receptor; *LOX-1*, lectin-like oxidized low-density lipoprotein receptor-1; *Lrp1*, low-density lipoprotein receptor-related protein 1; *LXRα*, liver X receptor alpha; *PCSK9*, proprotein convertase subtilisin/kexin type 9; *PLTP*, phospholipid transfer protein; *Pnlip*, pancreatic lipase; and *PPAR*, peroxisome proliferator-activated receptor.

In summary, EBN exhibits significant lipid-lowering effects through multiple pathways, including hepatic cholesterol metabolism, adipokine regulation, gut microbiota modulation, and estrogen-like activity (Table 1, Figure 2). Despite these promising findings, further research is needed to fully establish EBN’s efficacy and safety for dyslipidemia management. Future studies should focus on clinical trials in human populations to validate its lipid-lowering effects and optimal dosage. Long-term safety studies are also necessary to assess whether EBN interacts with conventional lipid-lowering therapies, particularly in individuals at high cardiovascular risk. Moreover, exploring personalized nutrition approaches, considering genetic variations in lipid metabolism, may help optimize EBN’s effectiveness for individualized dyslipidemia management.

## 4. Effects of EBN on Obesity

Obesity is characterized by excessive fat accumulation, which results from an imbalance between energy intake and expenditure [56]. Chronic low-grade inflammation, oxidative stress, and dysregulated lipid metabolism further contribute to obesity-related metabolic dysfunction [57]. While conventional treatments, including lifestyle modifications and pharmacotherapy, which are effective, there is growing interest in natural products with anti-obesity properties. Several studies have investigated EBN’s potential anti-obesity effects using diet-induced obesity models.

Findings from a study involving high-fat diet-induced obese C57BL/6J mice demonstrated that EBN supplementation for eight weeks significantly reduced body weight gain, fat mass, liver weight, and hepatic fat accumulation [42]. The anti-obesity effects of EBN were further supported by studies in high-fat diet-induced obese Sprague Dawley rats [45], as well as high-fat, high-cholesterol diet-induced obese New Zealand white rabbits and *Rattus norvegicus*, where EBN supplementation significantly reduced body weight gain [46,47]. Similarly, in ovariectomy-induced menopausal Sprague Dawley rats, EBN supplementation attenuated weight gain, suggesting that EBN’s estrogen-mimetic effects play a role in preventing menopause-associated obesity [48]. In contrast, some studies involving high-fat diet-induced obese Sprague Dawley rats reported no significant changes in body weight with EBN supplementation [30,31,44], indicating potential variations in EBN’s weight-regulating effects across different models.

The weight-regulating effects of EBN appear to be mediated through several metabolic pathways. One key mechanism is the modulation of the gut–liver axis, where EBN influences the gut microbiota composition, promoting the growth of beneficial bacteria involved in carbohydrate and fat metabolism. This microbial shift enhances energy expenditure and lipid breakdown, leading to reduced body fat accumulation [42]. Additionally, EBN enhances bile acid metabolism, which facilitates lipid digestion and excretion, thereby preventing excessive fat accumulation in the liver and adipose tissue [42]. Another potential mechanism is EBN’s ability to regulate lipid absorption and synthesis. Studies have shown that EBN supplementation reduces lipase activity, limiting dietary fat breakdown and absorption, ultimately leading to a reduced fat mass and weight gain [47]. Furthermore, EBN may exert hormonal effects, particularly in estrogen-deficient models, where its estrogen-like activity helps regulate the lipid metabolism and energy balance [48].

Several bioactive compounds in EBN have been proposed to contribute to its weight-lowering effects, including sialic acid, nucleobindin-2 (NUCB2), bakuchiol, and dehydrolindestrenolide. Studies have demonstrated that sialic acid supplementation improves energy expenditure and lipid metabolism, which may explain EBN’s observed effects on body weight reduction and fat mass regulation [42,58,59]. NUCB2, another bioactive protein identified in EBN, has been linked to appetite regulation and metabolic control. NUCB2 is a precursor of nesfatin-1, a peptide hormone that suppresses the appetite and promotes fat oxidation [60]. Studies have shown that NUCB2-deficient mice exhibit an increased body weight and fat accumulation, highlighting its role in obesity prevention [61]. Given that EBN contains NUCB2, its weight-regulating effects may, in part, be mediated through appetite suppression and energy balance modulation.

Meanwhile, bakuchiol and dehydrolindestrenolide, two active compounds identified in EBN, have been shown to inhibit lipase activity, reducing fat digestion and absorption. Studies have demonstrated that bakuchiol inhibits pancreatic lipase, limiting triglyceride breakdown and preventing excessive fat accumulation [62]. Similarly, dehydrolindestrenolide has been reported to reduce lipid uptake and accumulation in adipose tissues, reinforcing its potential anti-obesity effects [47]. The presence of these lipase-inhibitory compounds in EBN further supports its role in body weight regulation [47].

**Figure 3:**
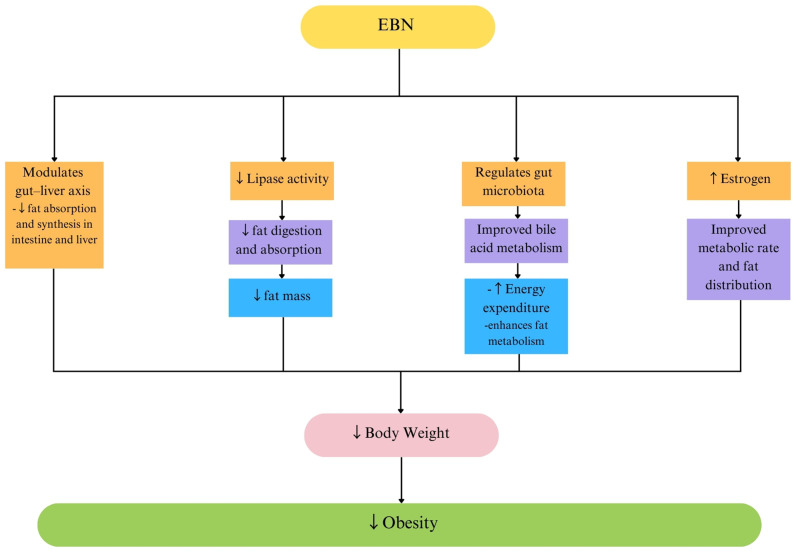
Effects of EBN on obesity. Abbreviations: ↑, increase; ↓, decrease; and EBN, edible bird’s nest.

In summary, EBN exhibits promising anti-obesity effects by reducing body weight gain, fat accumulation, and lipid absorption, potentially through its gut microbiota modulation, bile acid metabolism enhancement, and lipase inhibition (Table 2, Figure 3). Several bioactive compounds, including sialic acid, NUCB2, bakuchiol, and dehydrolindestrenolide, have been identified as potential contributors to its weight-regulating properties. While preclinical findings are encouraging, further human trials, and mechanistic studies are necessary to confirm EBN’s efficacy and safety as a functional food-based anti-obesity intervention. Additionally, long-term safety studies are needed to assess whether EBN interacts with conventional weight-loss medications or metabolic regulators. Moreover, exploring the gut microbiome’s role in EBN’s metabolic effects may provide insights into personalized dietary interventions for obesity management.

## 5. Effects of EBN on Diabetes Mellitus and Glucose Metabolism

Diabetes mellitus is a chronic metabolic disorder characterized by persistent hyperglycemia due to insulin resistance, pancreatic β-cell dysfunction, or both [63]. Insulin, a hormone produced by the β-cells of the pancreatic islets of Langerhans, plays a vital role in regulating blood glucose levels by facilitating the glucose uptake into the cells and modulating carbohydrate, lipid, and protein metabolism [63]. Insulin resistance, a hallmark of type 2 DM, is characterized by an impaired biological response to insulin in its target tissues, primarily the liver, muscle, and adipose tissue, leading to hyperinsulinemia as a compensatory mechanism and eventual pancreatic β-cell exhaustion [64]. In type 1 DM, pancreatic β-cell destruction results in an absolute insulin deficiency [65]. This dysfunction in insulin signaling leads to hyperglycemia and a low glucose tolerance, as the glucose uptake by peripheral tissues is impaired [64].

The progression of diabetes is closely linked to oxidative stress, chronic inflammation, and dysregulated insulin signaling, which collectively impair glucose metabolism and contribute to insulin resistance [66]. Prolonged hyperglycemia increases the production of ROS, leading to oxidative damage in pancreatic β-cells, which are particularly vulnerable to oxidative stress due to their low antioxidant capacity [67]. Additionally, chronic inflammation, primarily mediated by pro-inflammatory cytokines such as tumor necrosis factor-alpha (TNF-α), interleukin (IL-6) and IL-1β, further exacerbates insulin resistance by interfering with insulin receptor signaling, leading to a reduced glucose uptake in peripheral tissues [68].

Studies have demonstrated that EBN supplementation improves glucose metabolism and insulin sensitivity in obesity-induced insulin-resistant rats and mice [42,43,47], menopause-induced insulin-resistant rats [48], type 1 diabetic rats [69,70,71], and type 2 diabetic mice [72]. EBN exerts glucose-lowering effects by inhibiting α-glucosidase and α-amylase, two enzymes responsible for carbohydrate digestion and glucose absorption. In high-cholesterol, high-fat diet-induced obese rats, EBN supplementation significantly inhibited α-glucosidase and α-amylase activities, thereby reducing postprandial blood glucose levels [47]. EBN supplementation has also been shown to improve glucose tolerance and reduce fasting blood glucose (FBG), serum insulin levels, and the homeostatic model assessment of insulin resistance (HOMA-IR) values by upregulating key components of the insulin signaling cascade [31,47,72].

In db/db mice, a well-established model of type 2 DM, a hydrolyzed EBN supplementation for 28 days significantly reduced FBG levels, improved glucose tolerance, and increased the insulin expression in pancreatic β-cells [72]. Additionally, in diet-induced obese mice, EBN supplementation enhanced the secretion of glucagon-like peptide-1 (GLP-1), an incretin hormone that stimulates insulin release from pancreatic β-cells [42]. The improvement in the GLP-1 and insulin release may be contributed to by EBN’s modulatory effects on the circadian rhythm. The circadian rhythm plays a crucial role in adapting to daily energy demands by regulating appetite, insulin sensitivity, and glucose metabolism [72]. EBN influences liver circadian regulation by downregulating circadian locomotor output cycles kaput (*Clock*) and neuronal pas domain protein 2 (*Npas2*) gene expression and upregulating brain and muscle ARNT-like 1 (*Bmal1*), period circadian regulator (*Per)1*, and *Per2* gene expression [42]. By enhancing the circadian rhythm, pancreatic β-cell function, insulin expression, and GLP-1 secretion, EBN helps regulate blood glucose levels and support glucose homeostasis.

Furthermore, in insulin-resistant models, EBN increased the expression of insulin receptor (*InsR*), insulin receptor substrate (*IRS*) 1 and 2, phosphoinositide 3-kinase (*PI3K*), and protein kinase B (*Akt*), leading to an improved glucose uptake and utilization [43,48,72]. The phosphorylation of IRS proteins activates the PI3K/Akt signaling pathway, highlighting its importance in insulin’s metabolic functions [73]. In adipose tissue, insulin–Akt signaling enhances glucose utilization, inhibits gluconeogenesis, and promotes lipid biosynthesis. Meanwhile, in the liver, the activation of the PI3K/Akt pathway has been shown to reduce hepatic glucose production and glycogen breakdown while promoting glycogen and fatty acid synthesis for the storage and later use by other tissues [74]. Therefore, the activation of the PI3K/Akt pathway is crucial for insulin-mediated glucose transport and glycogen synthesis, and its impairment is a major contributor to insulin resistance [75]. EBN also downregulates the negative regulators of insulin signaling, such as mitogen-activated protein kinase 1 (*MAPK1*) and the inhibitor of nuclear factor kappa-B kinase subunit beta (*IKBKB*), which are known to interfere with insulin receptor activity [43]. By suppressing these negative regulators of insulin signaling, EBN restores the insulin responsiveness in peripheral tissues, hence improving glucose uptake and metabolism.

EBN also improves glucose metabolism by upregulating glucose transporters (GLUTs), which facilitate glucose’s entry into cells [48,72]. GLUT-2 and GLUT-4, two key glucose transporter isoforms, play a crucial role in regulating glucose uptake and disposal in the body. GLUT-2 is primarily expressed in glucose-sensing cells such as hepatocytes and pancreatic β-cells. During postprandial hyperglycemia, GLUT-2 facilitates continuous glucose uptake, triggering insulin secretion to maintain glucose homeostasis [76]. In contrast, the majority of the peripheral glucose uptake in adipose tissue and skeletal muscles relies on GLUT-4, an insulin-responsive glucose transporter regulated through similar signal transduction pathways [77]. Disruptions in the GLUT-4 function in muscles or adipose tissue impair insulin-mediated glucose uptake, contributing to insulin resistance and increasing the risk of diabetes development [78].

Studies have shown that EBN increases the expression of *GLUT-2* in the pancreas and liver and *GLUT-4* in skeletal muscles and adipose tissue, leading to an improved glucose uptake and clearance from circulation [72]. In ovariectomy-induced menopausal rats, EBN supplementation significantly increased *GLUT-4* gene expression, thereby improving glucose uptake in insulin-sensitive tissues [48]. Additionally, EBN modulates genes involved in hepatic glucose storage and utilization, including glucokinase (*GCK*), pyruvate kinase (*PK*), and ATP-sensitive potassium channel subunit (*KCNJ11*). These genes regulate glycogen synthesis and glycolysis, preventing excessive glucose accumulation in the circulation [43,48].

In obesity, excess body fat, especially visceral fat, plays a critical role in disrupting glucose metabolism and worsening insulin resistance in both the liver and peripheral tissues, ultimately increasing the risk of diabetes [79]. EBN supplementation reduced visceral fat accumulation by limiting lipid absorption and synthesis, while also promoting a bile acid balance through the modulation of the gut–liver axis, contributing to improved metabolic regulation [42]. Furthermore, obesity disrupts the balance of leptin and adiponectin, two key adipokines involved in energy homeostasis and insulin sensitivity [80]. Leptin, which regulates the appetite and metabolism, is often elevated in obesity, leading to leptin resistance, where the body no longer responds effectively to its appetite-suppressing effects [81]. Conversely, adiponectin, which enhances insulin sensitivity and fatty acid oxidation, is significantly reduced in obesity, contributing to insulin resistance and metabolic dysfunction [82]. This imbalance exacerbates glucose dysregulation, oxidative stress, and inflammation [83]. EBN has been found to increase adiponectin levels and reduce leptin levels, creating a metabolic environment that favors glucose homeostasis and insulin sensitivity [43].

The ability of EBN to mitigate oxidative stress and inflammation further supports its potential in diabetes management. Oxidative stress contributes significantly to diabetes progression by damaging β-cells, impairing insulin secretion, and reducing insulin sensitivity. EBN supplementation has been shown to reduce oxidative stress markers, such as malondialdehyde (MDA) and NADPH oxidase 4 (Nox4), while increasing the activity of key antioxidant enzymes, including superoxide dismutase (SOD), glutathione peroxidase (GPx), and catalase (CAT) [70,72]. In streptozotocin (STZ)-induced diabetic models, EBN supplementation lowered blood glucose levels and improved SOD levels [69,70]. These findings suggest that EBN provides a protective effect against oxidative damage in pancreatic β-cells, thereby preserving the insulin secretion capacity.

Furthermore, chronic inflammation plays a significant role in insulin resistance and β-cell dysfunction [84]. Pro-inflammatory cytokines such as TNF-α inhibit IRS1, which is crucial for insulin signaling [85]. EBN has been shown to reduce systemic inflammation by downregulating pro-inflammatory cytokines, such as TNF-α, IL-6, IL-1β; inducible nitric oxide synthase (iNOS); and nuclear factor kappa B (NFκB), while increasing anti-inflammatory mediators such as IL-10 and peroxisome proliferator-activated receptor gamma coactivator-1 alpha (PGC-1α) [47,72]. These findings suggest that EBN attenuates inflammation-induced insulin resistance. Additionally, EBN’s ability to reduce oxidative stress and inflammation is partially mediated through its role in enhancing the gut microbiota composition and modulating the gut–liver axis [42].

The antidiabetic effects of EBN can be attributed to its bioactive compounds, including sialic acid, lactoferrin, ovotransferrin, bakuchiol, and dehydrolindestrenolide [48]. Sialic acid has been reported to enhance insulin secretion, improve insulin sensitivity, and modulate the gut microbiota composition [86]. A study has demonstrated that sialic acid supplementation increases GLP-1 secretion, which enhances pancreatic β-cell function and promotes glucose-stimulated insulin release [42]. In diabetic models, sialic acid has also been shown to reduce fasting glucose levels and improve insulin signaling [87], findings that are consistent with the glucose-lowering effects of EBN. Similarly, lactoferrin and ovotransferrin, two glycoproteins present in EBN, have been linked to improved insulin signaling through the activation of the PI3K/Akt pathway, thereby enhancing glucose uptake and utilization in insulin-responsive tissues [48]. Additionally, these proteins exhibit antioxidant and anti-inflammatory properties [88], which contribute to the overall improvement of metabolic health.

Meanwhile, bakuchiol and dehydrolindestrenolide have been shown to act as α-glucosidase and α-amylase inhibitors, reducing carbohydrate digestion and glucose absorption in the intestines [47]. This mechanism helps prevent postprandial hyperglycemia, a key concern in diabetes management. Previous studies have also demonstrated that bakuchiol suppresses carbohydrate-digesting enzymes, leading to lower postprandial glucose excursions, while dehydrolindestrenolide has been reported to reduce oxidative stress and inflammation, further improving insulin sensitivity [89]. Given that EBN contains these bioactive compounds, its antidiabetic effects may be mediated, at least in part, through enzyme inhibition, antioxidant protection, and inflammation modulation.

**Figure 4:**
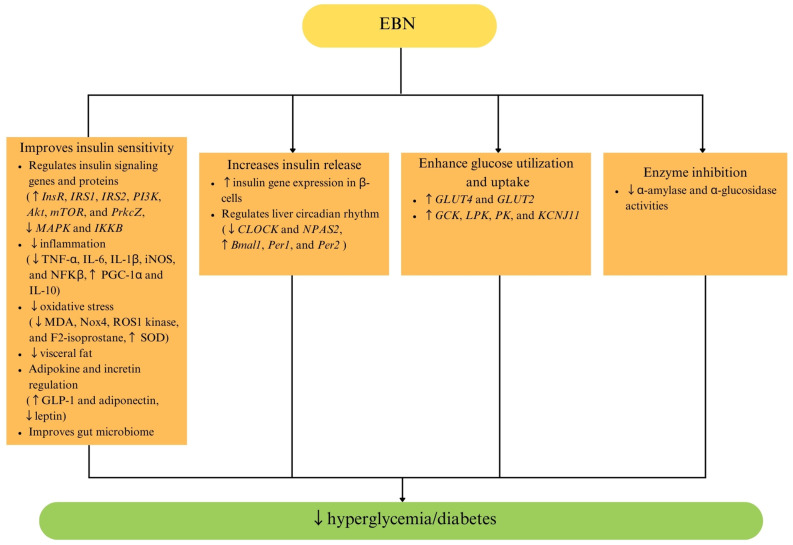
Effects of EBN on diabetes mellitus and glucose metabolism. Abbreviations: ↑, increase; ↓, decrease; *Akt*, protein kinase B; *Bmal1*, brain and muscle Arnt-like 1; *CLOCK*, circadian locomotor output cycles kaput; EBN, edible bird’s nest; *GCK*, glucokinase; GLP-1, glucagon-like peptide-1; *GLUT2*, glucose transporter type 2; *GLUT4*, glucose transporter type 4; *IKKB*, inhibitor of nuclear factor kappa-B kinase subunit beta; IL-1β, interleukin 1 beta; IL-6, interleukin 6; IL-10, interleukin 10; IL-6, *InsR*, insulin receptor; iNOS, inducible nitric oxide synthase; *IRS1*, insulin receptor substrate 1; *IRS2*, insulin receptor substrate 2; *KCNJ11*, potassium inwardly rectifying channel subfamily J member 11; *LPK*, liver pyruvate kinase; *MAPK*, mitogen-activated protein kinase; MDA, malondialdehyde; *mTOR*, mechanistic target of rapamycin; NFkβ, nuclear factor kappa B; Nox4, NADPH oxidase 4; *NPAS2*, neuronal PAS domain protein 2; *Per1*, period circadian protein homolog 1; *Per2*, period circadian protein homolog 2; PGC-1α, peroxisome proliferator-activated receptor gamma coactivator 1-alpha; *PI3K*, phosphoinositide 3-kinase; *PK*, pyruvate kinase; *PrkcZ*, protein kinase c zeta; ROS1 kinase, ROS proto-oncogene 1, receptor tyrosine kinase; SOD, superoxide dismutase; and TNF-α, tumor necrosis factor alpha.

In summary, EBN demonstrates significant antidiabetic effects by improving insulin sensitivity, enhancing glucose metabolism, reducing oxidative stress, and modulating inflammation (Table 3, Figure 4). Its bioactive compounds, including sialic acid, lactoferrin, ovotransferrin, bakuchiol, and dehydrolindestrenolide, contribute to its antidiabetic potential [42,43,47,72]. The concentration of sialic acid in EBN has been reported to range between 11.4 and 527 mg/kg [90], while lactoferrin and ovotransferrin have been identified at concentrations of 4.68 μg/mg and 10.23 μg/mg, respectively [43,52]. However, the specific concentrations of bakuchiol and dehydrolindestrenolide in EBN have not been documented in the literature. In addition to these compounds, EBN contains a notable total phenolic content, which is positively correlated with its antioxidant activity [19]. This suggests that phenolic compounds may also contribute to EBN’s overall biological activity, including its antidiabetic and broader cardiometabolic benefits. Despite promising preclinical evidence, further research is required to establish EBN’s efficacy in human diabetes management. Future studies should focus on conducting well-designed clinical trials to determine the optimal dosing, long-term metabolic effects, and safety profiles of EBN supplementation in patients with diabetes. Additionally, investigating the gut microbiome’s role in mediating EBN’s antidiabetic effects may also offer new perspectives for personalized dietary interventions tailored to individuals with diabetes.

## 6. Variability in EBN Composition

A notable aspect of EBN is the variability in its chemical composition depending on several factors, including the geographical origin, harvesting season, species of swiftlet, environmental conditions, and processing techniques [19]. Proximate analyses of EBN samples from Indonesia, Thailand, and Malaysia consistently show that crude protein constitutes the major component, ranging from 53.4% to 66.9% [91,92,93]. However, seasonal variations have also been observed, with some studies noting fluctuations in the protein content across different collection periods, possibly due to changes in the swiftlet diet or nesting environment [8]. The production environment, whether in natural caves or purpose-built swiftlet houses, also contributes to compositional differences. Cave nests, for example, may contain higher levels of minerals due to their exposure to bat guano and cave walls, while house nests may offer greater control over hygiene and safety standards [94,95].

The nutrient and bioactive composition of EBN is influenced not only by its origin and seasonal factors but also significantly by post-harvest preparation and processing methods. Raw unclean EBN typically undergoes a range of processing steps before it is deemed suitable for consumption, including soaking, feather removal, drying, shaping, and, in some cases, bleaching, enzymatic treatment, or sterilization. These steps can markedly alter the physicochemical properties and nutritional quality of the final product [95,96]. For instance, prolonged soaking can result in the leaching of water-soluble nutrients, such as sialic acid, amino acids, and certain trace minerals, reducing the bioactive potential of EBN. High-temperature drying or sterilization can denature thermolabile proteins, degrade antioxidant compounds, and potentially generate undesirable by-products [8,12].

Some commercial processors may employ hydrogen peroxide or bleaching agents to improve the visual appearance of EBN, especially to whiten lower-quality nests. However, these treatments can degrade bioactive components and introduce residual chemical contaminants if not thoroughly rinsed [97,98]. Enzymatic cleaning methods, which use food-grade proteases or carbohydrases, are increasingly adopted to preserve the glycoprotein content of EBN that contributes to its antioxidant, immunomodulatory, and anti-inflammatory properties [99]. These methods are considered gentler and more effective in preserving EBN’s nutritional profile compared to chemical or high-heat treatments. As such, the standardization of processing techniques is essential to minimize variability, maintain the bioactivity of key compounds such as sialic acid, and ensure consumer safety across different batches and sources of EBN.

## 7. Safety of EBN

EBN is a highly nutritional functional food that has been widely consumed for its numerous purported health benefits. While it is generally regarded as safe, a comprehensive evaluation of its safety profile remains essential, especially given the increasing global consumption of EBN. Currently, no formal toxicity study has been conducted to systematically assess the potential adverse effects of EBN consumption. This is a critical gap, as various factors, such as contamination, chemical composition, allergenicity, and regulatory standards, may influence the safety of EBN.

Contamination by bacteria, fungi, and mites poses a significant health risk, particularly in inadequately processed or improperly stored EBN [95]. Pathogens such as *Salmonella* sp., *Escherichia coli*, and *Staphylococcus aureus* have been detected in EBN, alongside fungi like *Aspergillus* sp. and *Penicillium* sp. [100]. Infestations by feather mites, house dust mites, and storage mites have also been reported [101]. These contaminants can lead to foodborne illnesses, including food poisoning, diarrhea, and even meningitis [95].

Chemical contamination is another area of concern. Nitrite and nitrate accumulation, which may occur during specific processing techniques or as a result of environmental exposure, has raised significant food safety alarms [102]. In 2011, the Chinese government banned the import of EBN products after detecting excessively high levels of nitrite, particularly in cave-collected samples, where concentrations reached up to 11,000 ppm. This incident prompted heightened scrutiny and led to stricter food safety regulations in China, particularly affecting major EBN-exporting countries, such as Indonesia and Malaysia [97]. In response, Chinese authorities implemented rigorous import requirements and monitoring systems to safeguard public health. EBN products destined for China must now comply with internationally recognized food safety standards, including Hazard Analysis and Critical Control Points (HACCPs) [103], Good Manufacturing Practices (GMPs) [104], and ISO 22000 [105]. Additionally, all imports must undergo mandatory sanitary inspections, quarantine protocols, and detailed traceability assessments regarding the product origin, composition, and processing [97]. To further strengthen regulatory oversight, the Chinese Academy of Inspection and Quarantine (CAIQ) issued two foundational documents that serve as benchmarks for EBN safety. The first outlines comprehensive hygiene practices, facility requirements, and quality control procedures for processing raw unclean EBN into raw clean EBN [106]. The second establishes certification procedures and inspection criteria to ensure that imported EBN meets China’s safety and quality standards [107]. These documents can be retrieved from the website http://ebn.caiq.org.cn/.

Additionally, although rare, EBN-related allergic reactions have been reported, presenting with symptoms such as angioedema, wheezing, urticaria, and abdominal cramps, though no fatalities have been documented [108]. The sources of these allergens may include the swiftlet’s saliva or feathers, insect remnants from the swiftlet’s diet, microorganisms and arthropods associated with the nests, or contaminants introduced during cleaning and processing [109]. EBN’s glycoprotein content may also trigger allergic reactions in sensitive individuals, particularly in individuals with pre-existing protein allergies [110]. Moreover, heavy metal contamination, including lead, mercury, and arsenic, has been detected in some EBN samples due to environmental pollution [102]. These findings underscore the importance of implementing standardized cleaning procedures, stringent contamination testing, and robust quality control measures to ensure the safety of EBN products across markets.

To scientifically classify EBN as a functional food appropriate for structured dietary interventions, several critical criteria must be met. These include the consistent and validated chemical characterization of bioactive components, a well-understood mechanism of action, the demonstrated bioavailability and metabolic stability of key compounds, and a reproducible clinical efficacy in human trials. Although the existing research supports the therapeutic potential of EBN, especially in metabolic and cardiovascular health, many of these scientific and regulatory prerequisites are still under active investigation. As such, although EBN holds great potential as a high-value functional food, further efforts in standardization, clinical validation, and regulatory alignment are essential before it can be confidently incorporated into structured dietary guidelines or therapeutic regimens.

## 8. Conclusions and Prospects

This review highlights the potential role of EBN as a functional food for reducing key CVD risk factors, particularly dyslipidemia, obesity, and DM. Preclinical evidence suggests that EBN exerts lipid-lowering effects, enhances glucose metabolism, and improves body weight regulation, all of which contribute to lowering the risk of atherosclerosis and cardiovascular complications. Through mechanisms such as hepatic cholesterol regulation, gut–liver axis modulation, insulin signaling enhancement, inflammation suppression, and oxidative stress reduction, EBN may provide a natural approach for CVD prevention and management. However, despite promising laboratory findings, clinical research on EBN’s cardiovascular benefits remains limited. Future studies should focus on human trials to confirm its efficacy, determine optimal dosages, and assess its long-term cardiovascular effects. Additionally, the role of EBN in hypertension management, another significant contributor to CVD, remains unexplored and requires further investigation. Understanding EBN’s potential interactions with conventional lipid-lowering and antidiabetic therapies could provide valuable insights into its role as a complementary intervention in cardiovascular health. Nevertheless, EBN should not be considered a substitute for conventional medical therapies in the management of dyslipidemia, obesity, and DM until more comprehensive clinical evidence becomes available.

## Figures and Tables

**Table 1:** Effects of EBN on dyslipidemia and lipid metabolism.

EBN Preparation and Source	EBN Dose	Experimental Model	Findings	Proposed Mechanism and Suggested Active Compound	Reference
EBN powder Terengganu, Malaysia	2.5% and 20% for 12 weeks	High-fat diet-induced obese Sprague Dawley rats	↓ TC, TG, LDLc ↑ HDLc ↓ oxLDL	EBN improves lipid profile by increasing adiponectin and reducing leptin levels. Suggested active compound: not stated.	[30]
EBN powder Terengganu, Malaysia	2.5% and 20% for 12 weeks	High-fat diet-induced obese Sprague Dawley rats	↓ TC, TG, LDLc ↑ HDLc ↓ oxLDL ↓ serum lipase	EBN improves hypercholesterolemia by regulating the expression of hepatic cholesterol metabolism genes (↑ *LDLR* and *CYP7A1*, and ↓ *PCSK9*, *APOB* and *HMGCR*). Suggested active compounds: sialic acid, lactoferrin, and ovotransferrin.	[44]
Full stew EBN and EBN stew extract Terengganu, Malaysia	500 mg/kg/day for 12 weeks	High-fat, high-cholesterol diet-induced atherosclerotic New Zealand white rabbits	↓ TC, TG, LDLc ↑ HDLc ↓ atherogenic indices	EBN ameliorates hypercholesterolemia by: - ↓ hepatic HMGCR levels - ↑ expression of genes for cholesterol uptake (*LDLR*, *LOX-1*, and *CD36*) - ↑ expression of genes for cholesterol efflux (*ABCA1*, *LCAT*, and *CYP7A1*) - ↑ expression of genes for cholesterol-sensing signaling (*LXRα* and *PPARγ*) Suggested active compound: sialic acid.	[46]
Fresh stewed EBN Xiamen, China	2777, 5555 and 11,111 mg/kg/day for 10 weeks	High-fat diet-induced obese C57BL/6J mice	↓ TC, TG, LDLc ↑ HDLc ↑ sialic acid content in LDL ↓ oxLDL	EBN improves lipid profile by: - regulating genes related to lipid absorption and metabolism in the intestine and the liver (*ABCA1*, *Fabp6*, *PLTP*, *PPAR*, *Lrp1*, *Ggt1*, *CYP2C66*, *CYP2C69*, *Pnlip*, *CLPS*, and *Elovl3*) - ↑ abundance of gut microbes related to carbohydrate and fat metabolism - ↑ hepatic bile acid excretion - ↓ intestinal secondary bile acid reflux Suggested active compound: sialic acid.	[42]
EBN powder Terengganu, Malaysia	2.5% and 20% for 12 weeks	High-fat diet-induced obese Sprague Dawley rats	↓ TC, TG, LDLc ↑ HDLc	The lipid-lowering mechanisms of EBN were not elucidated in the study. Suggested active compounds: sialic acid, lactoferrin, and ovotransferrin.	[43]
EBN powder Sarawak, Malaysia	1.5% and 3% EBN for 12 weeks	Ovariectomy-induced menopausal Sprague Dawley rats	↓ TC, TG, LDLc ↑ HDLc	EBN improves lipid profile by enhancing estrogen levels and through its estrogen-mimetic effects. Suggested active compound: not stated.	[48]
EBN powder East Java, Indonesia	In vitro: 50–250 µg/mL In vivo: 22.5 and 45 mg/kg/day for 6 weeks	High-cholesterol, high-fat diet-induced obese *Rattus norwegicus*	↓ TC, TG, LDLc ↑ HDLc	EBN improves lipid profile by reducing HMGCR and lipase activities. Suggested active compounds: bakuchiol and dehydrolinestrenolide.	[47]
EBN soup and extract Perak, Malaysia	843.2 mg/kg/day EBN soup or 6.5 mg/kg/day EBN extract for 6 weeks	High-fat diet-induced obese Sprague Dawley rats	↓ TC, TG, LDLc, VLDLc ↑ HDLc ↓ atherogenic indices	The lipid-lowering mechanisms of EBN were not elucidated in the study. Suggested active compound: sialic acid.	[45]

Abbreviations: ↑, increase; ↓, decrease; *ABCA1*, ATP-binding cassette transporter A1; *APOB*, apolipoprotein B; *CD36*, cluster of differentiation 36; *CLPS*, colipase; *CYP7A1*, cytochrome P450 family 7 subfamily A member 1; *Cyp2C66*, cytochrome P450 family 2 subfamily C member 66; *Cyp2C69*, cytochrome P450 family 2 subfamily C member 69; EBN, edible bird’s nest; *Elovl3*, elongation of very long chain fatty acids protein 3; *Fabp6*, fatty acid-binding protein 6; *Ggt1*, gamma-glutamyltransferase 1; HDLc, high-density lipoprotein cholesterol; HMGCR, 3-hydroxy-3-methylglutaryl-CoA reductase; *LCAT*, lecithin-cholesterol acyltransferase; *LOX-1*, lectin-like oxidized low-density lipoprotein receptor-1; *Lrp1*, low-density lipoprotein receptor-related protein 1; LDLc, low-density lipoprotein cholesterol; *LDLR*, low-density lipoprotein receptor; *LXRα*, liver x receptor alpha; oxLDL, oxidized low-density lipoprotein; *PCSK9*, proprotein convertase subtilisin/kexin Type 9; *PLTP*, phospholipid transfer protein; *Pnlip*, pancreatic lipase; *PPAR*, peroxisome proliferator-activated receptor; *PPARγ*, peroxisome proliferator-activated receptor gamma; TC, total cholesterol; and TGs, triglycerides.

**Table 2:** Effects of EBN on obesity.

EBN Preparation and Source	EBN Dose	Experimental Model	Findings	Proposed Mechanism and Suggested Active Compound	Reference
Fresh stewed EBN Xiamen, China	2777, 5555 and 11,111 mg/kg/day for 8 weeks	High-fat diet-induced obese C57BL/6J mice	↓ body weight gain ↓ body fat mass ↓ liver weight and fat accumulation ↑ energy expenditure	EBN attenuates obesity by: - ↓ lipid absorption and synthesis in the intestine and liver via the gut–liver axis - ↑ gut microbes related to carbohydrate and fat metabolism and energy metabolic pathways. - promoting bile acids metabolism Suggested active compound: sialic acid.	[42]
EBN powder Sarawak, Malaysia	1.5% and 3% for 12 weeks	Ovariectomy-induced menopausal Sprague Dawley rats	↓ body weight gain	EBN reduces weight gain during menopause by enhancing estrogen levels and through its estrogen-mimetic effects. Suggested active compound: not stated.	[48]
EBN powder East Java, Indonesia	In vitro: 50–250 µg/mL In vivo: 22.5 and 45 mg/kg/day for 6 weeks	In vitro enzyme inhibitory assays High-cholesterol, high-fat diet-induced obese *Rattus norwegicus*	↓ lipase activity in vitro ↓ body weight ↓ fat mass and obesity-associated proteins	EBN demonstrates anti-obesity effect by reducing serum lipase activity. Suggested active compounds: bakuchiol and dehydrolindestrenolide.	[47]
Full stew EBN and EBN stew extract Terengganu, Malaysia	500 mg/kg/day for 12 weeks	High-fat, high-cholesterol diet-induced New Zealand white rabbits	↓ body weight gain	The weight-lowering mechanisms of EBN are not elucidated in the study. Suggested active compounds: sialic acid and protein nucelobindin-2.	[46]
EBN soup and extract Perak, Malaysia	843.2 mg/kg/day EBN soup or 6.5 mg/kg/day EBN extract for 6 weeks	High-fat diet-induced obese Sprague Dawley rats	↓ body weight gain ↓ subcutaneous and visceral fat mass	The weight-lowering mechanisms of EBN are not elucidated in the study. Suggested active compound: sialic acid.	[45]
EBN powder Terengganu, Malaysia	2.5% and 20% for 12 weeks	High-fat diet-induced obese Sprague Dawley rats	No significant change in body weight with EBN supplementation.	-	[43]
EBN powder Terengganu, Malaysia	2.5% and 20% for 12 weeks	High-fat diet-induced obese Sprague Dawley rats	No significant change in body weight with EBN supplementation.	-	[44]
EBN powder Terengganu, Malaysia	2.5% and 20% EBN for 12 weeks	High-fat diet-induced obese Sprague Dawley rats	No significant change in body weight with EBN supplementation.	-	[30]

Abbreviations: ↑, increase; ↓, decrease; and EBN, edible bird’s nest.

**Table 3:** Effects of EBN on diabetes mellitus and glucose metabolism.

EBN Preparation and Source	EBN Dose	Experimental Model	Findings	Proposed Mechanism and Suggested Active Compound	Reference
Fresh stewed EBN Xiamen, China	2777, 5555, and 11,111 mg/kg/day for 8 weeks	High-fat diet-induced obese C57BL/6J mice	↓ FBG Improve glucose tolerance ↓ serum insulin ↓ HOMA-IR	EBN reduces insulin resistance by: - ↓ visceral fat - ↑ GLP-1 - ↓ inflammation (↓ IL-1β, IL-6 and TNF-α) - ↑ liver antioxidant capacity - improving the composition of the gut microbiota - ↑ insulin secretion by β-cells and circadian rhythm in the liver (through ↓ mRNA expression of *Clock* and *Npas2* and ↑ mRNA expression of *Bmal1*, *Per1*, and *Per2*) Suggested active compound: sialic acid.	[42]
EBN powder Terengganu, Malaysia	2.5% and 20% for 12 weeks	High-fat diet-induced insulin-resistant Sprague Dawley rats	Improve glucose tolerance ↓ insulin levels ↓ HOMA-IR	EBN reduces insulin resistance by: - ↑ insulin signaling (by upregulating the gene expression of *InsR*, *IRS2*, *PI3K*, *mTOR*, and *PrkcZ*, and downregulating the gene expression of *MAPK1* and *IKBKB* in the liver and adipose tissues) - ↑ glucose sensing by upregulating the gene expression of *Gck, Pk,* and *KCNJ11* in the liver and adipose tissues - regulating the adipokine levels (↑ serum adiponectin and ↓ serum leptin) - ↓ oxidative stress (↓ F2-isoprostane) Suggested active compounds: sialic acid, lactoferrin, and ovotransferrin.	[43]
Hydrolyzed EBN Selangor, Malaysia	75 and 150 mg/kg/day for 28 days	Type 2 diabetic db/db mice	↓ FBG Improve glucose tolerance ↓ serum insulin	The antidiabetic effects of EBN are mediated by: - ↑ insulin expression in the pancreas - ↑ expression of insulin signaling proteins (InsRβ, IRS1, PI3K, and Akt) in the liver and adipose tissues - ↑ *GLUT-2* expression in the pancreas and liver - ↑ *GLUT-4* expression in the liver and skeletal muscle - ↓ hepatic oxidative stress (↓ Nox4 and ↑ SOD) - ↓ inflammation (↓ serum TNF-α and IL-6, ↓ liver NFκB) Suggested active compound: sialic acid.	[72]
EBN powder Sarawak, Malaysia	1.5% and 3% for 12 weeks	Ovariectomy-induced menopausal Sprague Dawley rats	Improve glucose tolerance ↓ HOMA-IR	EBN prevents insulin resistance by: - regulating insulin signaling gene expression (↑ *InsR*, *IRS2*, and *PI3K*, *↓ MAPK* and *IKBKB*) - ↑ glucose uptake through ↑ *GLUT4* gene expression - ↑ gene expression for hepatic glucose storage and utilization (*GCK*, *LPK*, and *KCNJ11*) - ↓ hepatic oxidative stress (↓ MDA, ↑ SOD) Suggested active compound: not stated	[48]
EBN powder East Java, Indonesia	In vitro: 50–250 µg/mL In vivo: 22.5 mg/kg/day and 45 mg/kg/day for 6 weeks	In vitro enzyme inhibitory assays High-cholesterol, high-fat diet-induced obese *Rattus norwegicus*	↓ α-glucosidase and α-amylase activities ↓ blood glucose	The antidiabetic effect of EBN is contributed by: - α-glucosidase and α-amylase inhibition - ↓ oxidative stress (↓ ROS1 kinase, ↑ SOD) - ↓ inflammation (↓TNF-α and iNOS, ↑ PGC-1α and IL-10) Suggested active compounds: bakuchiol and dehydrolindestrenolide.	[47]
EBN aqueous extract Kalimantan, Indonesia	1, 10 and 100 mg/kg/day for 4 weeks	Streptozotocin-induced type 1 diabetic *Rattus norwegicus*	↓ blood glucose	EBN improves blood glucose of diabetic rats by reducing oxidative stress (↑ SOD). Suggested active compound: not stated.	[70]
EBN aqueous extract Kalimantan, Indonesia	1, 10 and 100 mg/kg/day for 4 weeks	Streptozotocin-induced type 1 diabetic *Rattus norwegicus*	↓ blood glucose	The glucose-lowering mechanisms of EBN are not elucidated in the study. Suggested active compound: not stated.	[69]
EBN aqueous extract Nakhon Si Thammarat, Thailand	75 and 150 mg/kg/day for 8 weeks	Streptozotocin-induced type 1 diabetic Wistar rats	↓ blood glucose	The glucose-lowering mechanisms of EBN are not elucidated in the study. Suggested active compound: not stated.	[71]

Abbreviations: ↑, increase; ↓, decrease; *Akt*, protein kinase B; *Bmal1*, brain and muscle ARNT-like 1; *Clock*, circadian locomotor output cycles kaput; EBN, edible bird’s nest; FBG, fasting blood glucose; *GCK*, glucokinase; GLP-1, glucagon-like peptide-1; *GLUT-2*, glucose transporter 2; *GLUT-4*, glucose transporter 4; HOMA-IR, homeostatic model assessment for insulin resistance; IL-1β, interleukin-1 beta; IL-6, interleukin-6; IL-10, interleukin-10; iNOS, inducible nitric oxide synthase; *InsR*, insulin receptor; *InsRβ*, insulin receptor beta subunit; *IRS1*, insulin receptor substrate 1; *IRS2*, insulin receptor substrate 2; *IKBKB*, inhibitor of kappa light polypeptide gene enhancer in B-cells, kinase beta; *KCNJ11*, potassium inwardly rectifying channel subfamily j member 1; *LPK*, liver pyruvate kinase; *MAPK*, mitogen-activated protein kinase; *MAPK1*, mitogen-activated protein kinase 1; MDA, malondialdehyde; *mTOR*, mammalian target of rapamycin; NFκB, nuclear factor kappa b; Nox4, NADPH oxidase 4; *Npas2*, neuronal pas domain protein 2; *Per1*, period circadian regulator 1; *Per2*, period circadian regulator 2; *PI3K*, phosphoinositide 3-kinase; *Pk*, pyruvate kinase; PGC-1α:,peroxisome proliferator-activated receptor gamma coactivator 1-alpha; *PrkcZ*, protein kinase c zeta; ROS1 Kinase, proto-oncogene tyrosine-protein kinase ros1; SOD, superoxide dismutase; and TNF-α, tumor necrosis factor-alpha.

## Data Availability

Data sharing is not applicable.
